# A New System for Assessing Visual Disability Using a Digital Visor

**DOI:** 10.3390/jcm9041086

**Published:** 2020-04-11

**Authors:** Raffaele Sangiuolo, Filippo Amore, Mauro Bacci, Paolo Brusini, Filippo Cruciani, Giacomo Gualtieri, Massimo Lancia, Giulia Sangiuolo, Mario Sangiuolo

**Affiliations:** 1 Italian Foundation of Digital and Robotic Ophthalmology (F.I.O.D.E.R.), 84134 Salerno, Italy; raffaelesangiuolo@tiscali.it; 2 Italian National Centre of Services and Research for the Prevention of Blindness and Rehabilitation of the Visually Impaired—WHOCC, IAPB Italy Onlus—FPG IRCCS, 00168 Roma, Italy; f.amore@iapb.it (F.A.); filippo.cruciani@uniroma1.it (F.C.); 3 Legal Medicine, Forensic Sciences, and Sport Medicine Section, University of Perugia, 06100 Perugia, Italy; mauro.bacci@unipg.it (M.B.); massimo.lancia@unipg.it (M.L.); giulia.sangiuolo@gmail.com (G.S.); 4 Department of Ophthalmology, “Città di Udine” Health Clinic, 33100 Udine, Italy; 5 Department of Medical Sciences, Surgery and Neurosciences, Section of Legal Medicine, Santa Maria alle Scotte University Hospital of Siena, 53100 Siena, Italy; drgiacomogualtieri@gmail.com; 6 Department of Ophthalmology, NHS Latina Pontino Center University of Roma “La Sapienza”, 04100 Latina, Italy; sangiuolomario@yahoo.it

**Keywords:** visual residual coefficient, visual disability, digital visor

## Abstract

Background: Considering the lack of universally accepted visual requirements for driving and for defining various grades of visual disability, the aim of this study is to propose a new method that provides a numerical score resulting from a combined assessment of the visual field and visual acuity loss obtained using a digital technology visor. Methods: This study presents a new system for calculating the percentage of visual disability by combining binocular visual acuity and binocular visual field assessments. A new Global Vision Evaluation System digital technology visor uses standardized, reproducible criteria to produce well-defined, numerically expressed test results. Through a specific algorithm, the device produces a numerical value expressing the percentage of visual disability. Results: Eighty-six subjects with various types of visual impairment underwent visual acuity and visual field test examinations carried out employing both traditional methods and the new digital visor. The two methods provided homogeneously similar results regarding the positioning of the subjects on the visual disability scale. Conclusions: The new digital visor seems to be a valid method to ensure that visual disability assessments are more homogeneous and reliable, and that, consequently, the resources available for this purpose are more fairly distributed.

## 1. Introduction

A recent transnational review of visual driving standards [1] has reported that the existing literature does not provide uniform driving standards, as there is no agreement over visual requirements for driving and the methods to be used to assess this ability. However, in over 130 countries worldwide, motorists are given permission to drive abroad without further tests if their domestic licence is valid. According to Casson and Racette [2] both visual driving standards and valid and reliable assessment tools are needed. Currently, many driver-licensing screening assessments worldwide are based on visual acuity (VA) and visual field (VF) examination, but the testing methods are not standardized, although a large number of studies have tried to assess the validity and reliability of the tools used to measure VA in normal subjects, or VA in specific populations [3,4,5,6,7,8,9].

Therefore, the role of vision in driving must be further investigated in order to identify what the requirements are for safe driving and to standardize vision assessment methods. Hence the importance of providing guidance on specific methods for determining a valid tool to assess visual driving skills.

Another important issue is the quantification of visual impairment, which is closely related to driving performance [10,11,12,13,14]. No homogeneous rules or standardized methods for quantifying visual disability are in use in different countries, or even within the same nation, and there are no clear and well-defined methods available to obtain such a quantification. 

In Italy, the 2015 National Social Security Institute (INPS) financial management audit report, published by the Court of Auditors, highlighted that “legislation aspiring to rationalize the procedure has not met the legislator’s aims of simplification and speed”. The main critical areas that produced what the Court of Auditors described as dissatisfying results were a) the lack of nationwide homogeneity of the assessment systems of the variously located commissions due to the absence of well-codified, standardized systems and instruments; b) the non-use of combined binocular visual field and visual acuity assessment to define visual disability [15,16]. This latter point is particularly important, considering that various countries use differing methods to calculate visual disability. In Italy, for example, central visual impairment is assessed by determining the visual acuity, whereas the peripheral impairment is quantified using a specific visual field testing method (Campo Visivo (CV)% by Gandolfo and Zingirian), according to the Italian law no. 138/2001. The situation is even more confusing with regards to driver licensing as most jurisdictions do not specify any method of visual field assessment or provide any clinical indications. 

The points briefly highlighted above provide sufficient evidence to understand the need for setting new guidelines for those affected by visual impairment in order to ensure a good level of visual capacity assessment, homogeneously implemented over the entire national territory, which employs a well-codified procedure using the combined examination of binocular visual field and binocular visual acuity. Reliable tools are needed to make individualized assessments of the actual impact of vision loss on driving ability since driving requires detection of complex stimuli and simultaneous use of both peripheral and central vision.

The aim of this study is to formulate a proposal for the determination of the percentage of visual disability through the elaboration of a numerical score resulting from the combined assessment of the binocular visual field and visual acuity obtained by means of a digital technology visor. This is a preliminary investigation, which precedes a future validation study concerning its potential to supply valid and reliable measurements to be used for calculating visual disability both for medico-legal purposes, and for driving license requirements.

## 2. Study Design

The proposed study is a prospective, observational, non-randomized study conducted on a group of subjects who were visually impaired or had fragile vision.

## 3. Material and Methods

Eighty-six subjects aged between 14 and 80 (mean 62.7 ± 15.4) with binocular visual acuity with the best optical correction between 0.05 and 1 were enrolled in this study. Of these, 50 were affected by a defect in the central visual field (within 30°), 11 in the peripheral field (outside 30°), and 25 had mixed defects.

Patients were excluded if they were not able to undertake the required tests or had the following characteristics: clinically relevant cataracts; evident cognitive alterations reducing patient reliability; or lack of consent.

All the clinical tests were performed at the Italian National Center of Services and Research for the Prevention of Blindness and Rehabilitation of the Visually Impaired at Gemelli Hospital in Rome. Patients were assessed in the period between November 2018 and March 2019. 

All subjects gave written informed consent before being included in the study. No protocol was submitted to an Ethics Committee prior to initiating the study, as it is an observational study that does not assign treatment to the participants. The investigations were carried out following the guidelines of the Declaration of Helsinki. All subjects received detailed information about the aims of the study. Participation was voluntary and subjects were free to withdraw consent at any time without consequences to their care.

The clinical tests were performed on subjects affected by maculopathy, inherited retinal degenerations, glaucoma, diabetic retinopathy, and degenerative myopia [17].

All patients underwent a full ophthalmic examination including: (1) measurement of VA using Early Treatment Diabetic Retinopathy Study (ETDRS) charts. (2) VF testing using an automated Humphrey Field Analyzer II perimeter (Carl Zeiss Meditec Inc., Dublin, CA, USA) with the Custom CV% binocular program created by Gandolfo and Zingirian (1991) [18]. This test, recommended by Italian law #138/3, April 2001, employs 100 test points strategically distributed up to 60° (64 points within the central 30°) according to a three-zone strategy. The percentage result (perimetric residual) is obtained by adding the number of points seen at the first presentation (value =1) to those seen only at maximum light intensity (value = 0.5). (3) Binocular VA and binocular VF assessment performed using the Global Vision Evaluation System (GVES) digital technology visor [19].

The proposed system uses a GVES digital visor with a specific software program for the assessment and classification of disability in visually impaired subjects affected by various pathologies leading to a central, peripheral, or mixed visual field deficit. The GVES digital visor uses virtual reality and resembles an auto refractor. Its maximum resolution is 2016 × 1200 pixels. The refresh rate is 90 Hz. The patient leans their head onto the headrest and uses a specific button to signal the perception of a stimulus during the visual field test. The maximum visual field amplitude is 110 degrees horizontally. The digital visor is able to perform the following tests: VA, binocular and monocular VF test, stereopsis, contrast sensitivity, glare recovery time, color sense. Considering the various test results, the instrument automatically elaborates a global visual index (GVI) that is the numerical expression of overall visual quality [20]. A special algorithm in the software specifically assesses visual disability by integrating the values obtained from the binocular VF and VA assessments to calculate the visual disability coefficient (VDC) automatically.

The measurement of binocular VA is performed by the digital visor using a scale of 13 increasing measurement levels, which are as follows (the visual acuity level scores are in brackets): 1/50 (2); 1/30 (3,33); 1/20 (5); 0.1 (10); 0,2 (20); 0.3 (30); 0.4 (40); 0.5 (50); 0.6 (60); 0.7 (70); 0.8 (80); 0.9 (90); 1 (100).

The visual acuity measurement employs an external display for values ranging between 2/20 and 20/20, according to the Snellen chart. For lower visual acuities, the measurement is performed using the internal display, where the dimension of the various letters is virtually determined through a computer calculation. 

**Figure 1:**
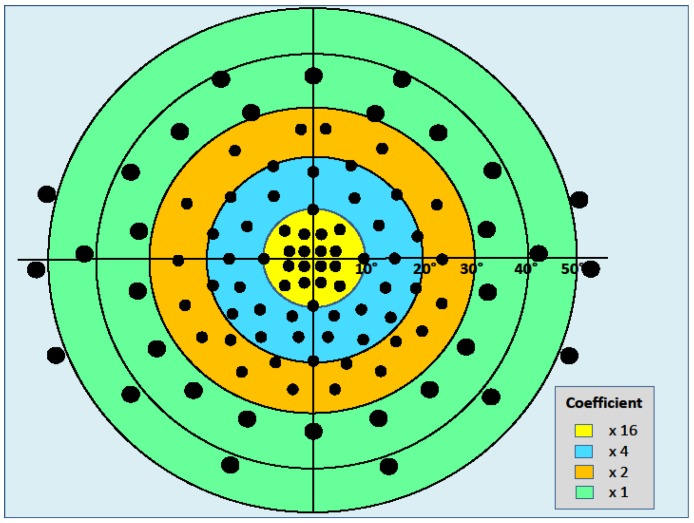
Test point distribution for binocular visual field (VF) testing with different coefficient scores.

Calculation of the residual binocular VF is carried out through the presentation of 100 target points according to the pattern shown in Figure 1. Each point is assigned a relative-importance coefficient, established in a previous study (unpublished data), differentiated according to its location, whose value increases centripetally (see Figure 1). In the area within the central 10 degrees, each point seen receives a score of 16, considering that the central field is of the upmost importance for reading and for many visual tasks. Between 10 and 20 degrees, the allocated score is 4, and between 20 and 30 degrees, the score given is 2. Points outside 30 degrees are scored as 1. The maximum possible overall score, obtained by multiplying the number of points by their respective relative-importance coefficient, is equal to 504.

The visual field parameters are similar to those used for standard automated perimetry. Within the 30 degree area, a Goldmann III target (6 mm^2^) was used, whereas outside this area the stimulus size was increased to 16 mm^2^ (IV target). The background luminance was set at 31.5 apostilb (Table 1).

The contrast between the targets and background was increased with respect to the standard values and differentiated based on age range due to the physiological variation in perception threshold (Table 2).

To ensure good central fixation, the subject examined was asked to recognize a central symbol (numbers or letters) presented at randomized intervals for 2”. During the period of presentation of this image, no stimuli were presented.

The percentage value of the residual VF is equal to the total number of points perceived, multiplied by their coefficient percentage, divided by 504, and then multiplied by 100.

For example, if a subject perceives points whose overall value is 88, his residual visual field is: 88/504 × 100 = 17.46%

### 3.1. Calculation of Visual Disability Percentage

To calculate the residual visual coefficient (RVC), the binocular VA value expressed in hundredths is multiplied by the residual binocular VF percentage using the following formula: VA × VF%/100. For example, a subject with a binocular VA of 2/10 (=20/100) and a binocular VF score of 88 (88/504 × 100 = 17.46%) will have a CVR of 20 × 17.46/100 = 3.5%

The visual disability coefficient (VDC) is then calculated using the following formula: 100 − RVC (considering the percentage of a perfectly normal subject as 100). Therefore, the subject in the preceding example would have a VDC of 100 − 3.5 = 96.5%.

Thanks to the algorithm incorporated into its software, the digital visor automatically calculates and displays on its screen the numerical value defining the disability percentage of the subject under examination.

### 3.2. Statistical Analysis

The characteristics of the 86 subjects enrolled in the study were initially examined using descriptive methods comparing their mean age, visual acuity, visual field, and disability score according to the type of visual defect (central, peripheral, or mixed). The null hypothesis of mean values being equal was tested using the non-parametric Kruskal–Wallis and Mann–Whitney tests, since the violation of normality was detected by the Kolmogorov–Smirnov test.

Given that the objective of the study was to verify whether visual acuity and visual field measurements obtained using a digital visor were comparable to traditional methods, two generalized linear models were used with the aim of assessing the continuous response variables (TMVF: traditional measure of visual field; TMVA: traditional measure of visual acuity) through the measures of VF and VA obtained by the digital visor (GVES_VF and GVES_VA). We used this method for its robustness against normality violations and because it enables the estimation of parameters by controlling for other predictors. Age (AGE) and defect type (DT) were used in the models in order to control the estimation of GVES_VF and GVES_VA for these individual characteristics. AGE was excluded from the second model because of its multicollinearity with the other predictors.

TMVA = β_0_ + β_1_ AGE + β_2_ DT + β_3_ GVES_VA + E (1)

TMVF = β_0_ + β_1_ DT + β_2_ GVES_VF + E (2)

Each model was tested for basic assumptions, and multicollinearity was assessed comparing Type I and Type III sum of squares estimations of effects. The significance of the omnibus test was checked to assess the predictive power of the models.

The comparability of the disability percentage obtained employing the two methods was assessed using the Pearson’s r correlation index. All statistical analyses were performed using SPSS-IBM v23 software. The significance level was set at *p* < 0.05.

## 4. Results

Of the 86 subjects examined, 50 presented a central visual defect, while 11 subjects showed peripheral impairment. In 25 cases, the defect was mixed. The general characteristics of the visual defect groups showed considerable heterogeneity (Table 3); mean age and mean visual acuity in patients tested traditionally and by digital visor revealed no significant differences among defect types. In contrast, significant differences were found among groups for VF measured using the automated Humphrey Field Analyzer II perimeter with the custom VF% binocular program (defined as the traditional method), and for VF measured with the visor (K-W: both with *p* < 0.000). The disability scores obtained by traditional methods and disability scores calculated by the visor showed significant differences among defect types as well (respectively: K-W, *p* < 0.000 and *p* < 0.004).

The first model expresses the level of VA obtained by the traditional method through AGE and DT as control variables and the measures of VA obtained by the digital visor (GVSE_VA) as predictor. The estimate of the main effects model provided a good fit: The omnibus test is significant (*p* < 0.004), and the accurate predictive power is confirmed by the ratio between the measure of deviance and its degrees of freedom (deviance/df = 0.032). As shown in Table 4, the absence of multicollinearity was detected by the consistency of Type I and Type III sum of squares statistics. Controlling for AGE and DT, which had non-significant main effects, GVES_VA represented a significant predictor of VA as measured using traditional EDTRS measurement (*p* < 0.001).

Analysing the estimates of the parameters (Table 5), none of the categories of DT were significant, although the “Mixed” class showed a borderline *p*-value (*p* < 0.060). The positive sign of GVES_VA’s parameter (*β* = 0.201) showed a direct relationship: increasing values of this predictor result in increasing levels of the dependent variable (VA obtained by the traditional method). Wald’s chi-square of this predictor was significantly higher than that of the others in the model.

The two binocular VA assessment methods seem to be globally equivalent. It should be noted however that for VA < 0.1 (12 cases) the correlation was not sufficient to affirm that the two methods offered the same result (Pearson’s r = 0.251; *p* < 0.457). Further research is needed to better understand the reason for this lack of correlation in patients with extremely low VA.

In regard to the VF assessment (Table 6 and Table 7), the model was estimated excluding the variable AGE, due to its multicollinearity with the other predictors. The estimation of the model proposed is significantly different from that of the model with intercept only (omnibus test: *p* < 0.000), even though the ratio between the measure of deviance and its degrees of freedom is very high (deviance/df = 331.032). Predictors showed a lack of multicollinearity. A significant main effect was found for DT (*p* < 0.000), and, controlling for this variable, the measurement of VF through the digital visor was significant as well (*p* < 0.000).

Central visual defect provided a significant parameter (*β* = 37.486; *p* < 0.000), underlining that only this defect type, if compared with mixed and peripheral defects, can affect the traditional measurement of VF. In accordance with the previous model, the positive sign of the GVES_VF’s parameter proved the direct and significant relationship with the dependent variable (*β* = 0.528: *p* < 0.000) and its Wald’s chi-square was the highest among the predictor’s values.

Finally, visual disability calculated using the binocular VA and VF assessment measured by the digital visor and elaborated by its incorporated algorithm was compared to the visual disability percentage obtained by combining the results of exams carried out using traditional methods. The mean disability percentage measured using GVES was 72.97%, while that obtained using traditional methods was 73.02%, a difference that was not statistically significant (M-W: *p* < 0.679).

The two methods provided homogeneous results regarding the positioning on the visual disability scale. This comparison was performed on a subset of 67 patients that presented only visual disability; 21 patients were excluded since their disability index was affected by other non-visual disabilities. The Pearson’s r correlation index between the two parameters was 0.685 (*p* < 0.013), showing an excellent degree of interchangeability between the traditional-method disability score and the disability score obtained using the digital visor.

## 5. Discussion

The issue of the validity and reliability of methods for measuring visual acuity and visual field for visual disability assessment and for driver licensing is still very contentious in the literature. Tan and colleagues [15] observed that the results of the measurement of visual field are affected by varying degrees of unreliability, although their conclusions have been criticized by other authors who object that their findings are not supported by a robust methodological framework [16].

The results of this preliminary study suggest that a digital visor has an encouraging potential to overcome the problems related to the imprecision of the VA and VF measurements and lack of standardized rules for determining functional abilities.

Concerning the VA measurement, the results obtained with the digital visor and traditional methods are very similar, but the use of a standardized and reproducible testing method, which guarantees a more accurate assessment, would overcome some biases related to methods currently in use in clinical practice, such as test illumination and distance.

With regards to the VF examination, the coefficient scores that were used are of course arbitrary and debatable. However, the results of this study seem to suggest they accurately quantify the severity of visual field defects. Moreover, in this case, the use of a standardized testing technique, accepted as a gold standard, would represent an important step forward for a precise, homogeneous and automatic quantification of VF loss, which is not easily obtainable using traditional visual field testing techniques, such as the Esterman’s grids [21] or the CV% [18].

Another important advantage in using a digital visor is that all tests (including others not addressed in this study, such as contrast sensitivity or color vision) can be performed with the same instrument without any need to move the subject, thus saving time and space.

In conclusion, the two different methods produced test results characterized by good homogeneity, except for low visual acuity scores, which are likely to be more accurately assessed with the digital visor, although further studies are needed to confirm this hypothesis. These data implicitly confirm the validity of both systems of assessment.

There are, however, several reasons for preferring the use of a digital technology visor over traditional methods. Firstly, test results are numerically defined and standardized with no interference from environmental factors (luminosity, test distance, instrument component wear, etc.,). Secondly, the disability percentage, calculated from the residual visual coefficient, can be expressed immediately at the end of the examination, thus producing a fast and simplified evaluation procedure. Thirdly, it would be possible to institute a nationwide well-codified homogeneous assessment system based on a single standard testing instrument instead of the numerous non-standardized instruments currently in use. This would overcome the problems reported by the Court of Auditors, which are mainly related to the lack of homogeneity of the assessment systems among the various commissions located nationwide.

In sum, adopting the digital visor would provide standardized and reproducible examination results as it produces an automated numerical expression of the percentage of visual disability and this would promote greater uniformity of judgment in centers tasked with assessment.

The next important step will be to determine how various grades of visual disability affect driving ability, so as to produce a standardized test able to reliably distinguish subjects with sufficient visual capacity from those who should be excluded from driving for safety reasons. This goal can be met by using a driving simulator to challenge subjects with various driving conditions [22,23].

## 6. Conclusions

Our experimental results show that the use of a digital visor is a valid method for ensuring that visual disability assessment is more homogeneous and reliable, and that, consequently, the resources available for this purpose are more fairly distributed.

## Figures and Tables

**Table 1:** Test parameters used for VF examination.

(1) Background luminance: 31.5 asb
(2) Maximum luminance of stimulus (0 dB): 2500 asb
(3) Size of stimuli: target III (4 mm^2^) within 30°; target IV (16 mm^2^) between 30° and 55°
(4) Presentation of stimuli: randomized sequence; presentation interval variable between 0.6”and 1.8”
(5) Repetition of points not seen at first presentation
(6) Presentation time: 0.6”
(7) Response time: 1.5”
(8) Correction coefficient in percentage used to emphasize test points closer to the center
(9) Focusing at infinity (no need for near correction)

**Table 2:** Test stimuli luminance according to the patient’s age and point location.

	Within 10°	Between 11° and 20°	Between 21° and 30°	Outside 30°
0–40 years	12%	18%	36%	60%
	9.6 lux	14.4 lux	28.8 lux	48 lux
	9.8 dB	11.5 dB	14.5 dB	16.8 dB
41–60 years	15%	30%	60%	82%
	12 lux	24 lux	48 lux	65.6 lux
	10.8 dB	13.8 dB	16.8 dB	18.1 dB
>60 years	18%	36%	72%	90%
	4.4 lux	28.8 lux	57.6 lux	72 lux
	11.5 dB	14.5 dB	17.5 dB	18.5 dB

**Table 3:** Mean and standard deviation in subjects with central, mixed, or peripheral defects by age and by scores obtained employing traditional methods or the visor.

Type of Defect	Central (*n* = 50)	Mixed (*n* = 25)	Peripheral (*n* = 11)	K-W Statistic	*p*
Age	64.9 ± 15.8	62.7 ± 13.5	52.8 ± 14.8	K-W_(2)_ = 5.432	0.066
Traditional-method scoring					
ETDRS binocular VA	0.44 ± 0.9	0.43 ± 0.9	0.99 ± 2.3	K-W_(2)_ = 1.813	0.404
VF% score	97.0 ± 126.2	34.90 ± 21.8	32.46 ± 50.6	K-W_(2)_ = 44.173	0.000 ^a^
Current Disability Score	79.2 ± 16.8	91.1 ± 11.1	93.7 ± 10.7	K-W_(2)_ = 19.657	0.000 ^b^
GVES. scoring					
Binocular visual acuity	0.28 ± 0.3	0.30 ± 0.4	0.31 ± 0.2	K-W_(2)_ = 0.631	0.730
Residual visual field	72.6 ± 27.9	43.6 ± 28.8	26.8 ± 23.1	K-W_(2)_ = 25.596	0.000 ^c^
Visual Disability Coefficient	81.4 ± 13.8	123.8 ± 178.9	91.7 ± 11.0	K-W_(2)_ = 11.252	0.004 ^d^

K-W: Kruskal-Wallis; ETDRS: Early Treatment Diabetic Retinopathy Study; VF: Visual Field; GVES.: Global Vision Evaluation System; VA: visual acuity; VF: visual field. Comparisons between groups (Mann–Whitney U): a) Central vs. Mixed: M-W U = 125.000, *p* < 0.000; Central vs. Peripheral: M-W U = 28.500, *p* < 0.000; Mixed vs. Peripheral: M-W U = 72.500, *p* < 0.026. b) Central vs. Mixed: M-W U = 321.000, *p* < 0.001; Central vs. Peripheral: M-W U = 89.500, *p* < 0.001; Mixed vs. Peripheral: M-W U = 95.000, *p* < 0.151. c) Central vs. Mixed: M-W U = 281.500, *p* < 0.000; Central vs. Peripheral: M-W U = 61.500, *p* < 0.000; Mixed vs. Peripheral: M-W U = 88.000, *p* < 0.093. d) Central vs. Mixed: M-W U = 388.000, *p* < 0.008; Central vs. Peripheral: M-W U = 135.000, *p* < 0.009; Mixed vs. Peripheral: M-W U = 121.000, *p* < 0.571.

**Table 4:** Test for the effects of the model with the traditional measure of VA as dependent variable; Type I and Type III sum of squares statistics.

		Type I SS		Type III SS	
Source	Df	Wald’s Chi-Square	*p*	Wald’s Chi-Square	*p*
Intercept	1	193.571	0.000	8.753	0.003
AGE	1	1.520	0.218	0.2106	0.650
DT	2	3.586	0.166	3.887	0.143
GVES_VA	1	11.741	0.001	11.741	0.001

SS = Sum of Squares, Df = degrees of freedom, DT = defect type, AGE = age, GVES_VA = measure of VA obtained by the digital visor.

**Table 5:** Parameter estimates of the model with the traditional measure of VA as dependent variable.

Parameter	*β*	Standard Error	Wald’s Chi-Square	Df	*p*
Intercept	0.308	0.089	12.073	1	0.001
AGE	−0.001	0.001	0.206	1	0.650
DT - Central	−0.062	0.056	1.102	1	0.294
DT - Mixed	−0.120	0.064	3.525	1	0.060
DT - Peripheral	0.00	-	s-	-	-
GVES_VA	0.201	0.056	11.741	1	0.001

Df = degrees of freedom, DT = defect type, AGE = age, GVES_VA = measure of VA obtained by the digital visor.

**Table 6:** Test for the effects of the model with the traditional measure of VF as dependent variable; Type I and Type III sum of squares statistics.

		Type I SS		Type III SS	
Source	Df	Wald’s Chi-Square	*p*	Wald’s Chi-Square	*p*
Intercept	1	994.523	0.000	21.988	0.000
DT	2	172.926	0.000	49.287	0.000
GVES_VF	1	55.921	0.000	55.921	0.000

VF = Visual Field, Df = degrees of freedom, DT = defect type, GVES_VF = measure of VF obtained by the digital visor.

**Table 7:** Parameter estimates of the model with the traditional measure of VF as dependent variable.

Parameter	*β*	Standard Error	Wald’s Chi-Square	Df	*p*
Intercept	3.991	5.681	0.494	1	0.482
DT - Central	37.486	6.744	30.893	1	0.000
DT - Mixed	7.894	6.537	1.459	1	0.227
DT - Peripheral	0.00	-	-	-	-
GVES_VF	0.528	0.071	55.921	1	0.000

VF=Visual Field, Df = degrees of freedom, DT = defect type, GVES_VF = measure of VF obtained by the digital visor.
